# Updates on the Care of Cloacal Exstrophy

**DOI:** 10.3390/children11050544

**Published:** 2024-05-02

**Authors:** Claire A. Ostertag-Hill, Patrick T. Delaplain, Ted Lee, Belinda H. Dickie

**Affiliations:** 1 Department of Surgery, Boston Children’s Hospital, 300 Longwood Ave., Boston, MA 02115, USA; claire.ostertag-hill@childrens.harvard.edu (C.A.O.-H.); patrick.delaplain@childrens.harvard.edu (P.T.D.); 2 Department of Urology, Boston Children’s Hospital, 300 Longwood Ave., Boston, MA 02115, USA; ted.lee@childrens.harvard.edu

**Keywords:** cloacal exstrophy, OEIS, continence, bladder

## Abstract

Cloacal exstrophy is the most severe congenital anomaly of the exstrophy–epispadias complex and is characterized by gastrointestinal, genitourinary, neurospinal, and musculoskeletal malformations. Individualized surgical reconstruction by a multidisciplinary team is required for these complex patients. Not infrequently, patients need staged surgical procedures throughout childhood and adolescence. Following significant improvements in medical care and surgical reconstructive techniques, nearly all patients with cloacal exstrophy now survive, leading to an increased emphasis on quality of life. Increased attention is given to gender identity and the implications of reconstructive decisions. Long-term sequelae of cloacal exstrophy, including functional continence and sexual dysfunction, are recognized, and many patients require ongoing complex care into adulthood.

## 1. Introduction

Cloacal exstrophy (CE) is a rare congenital malformation characterized by exstrophy of the urinary bladder and cecal plate through an abdominal wall defect, anal atresia, colonic hypoplasia, omphalocele, and anomalous genitalia [1]. It is known as OEIS syndrome (Omphalocele–Exstrophy–Imperforate Anus–Spinal Anomalies Complex) in the current nomenclature. Patients can have all or some of the issues involved, but it is one of the most complex congenital pelvic malformations in a spectrum of anomalies known as the exstrophy–epispadias complex (EEC), which also includes epispadias and bladder exstrophy [1,2]. In comparison, bladder exstrophy typically does not impact the gastrointestinal tract and is rarely associated with spinal anomalies. Epispadias comes in a wide spectrum (from glans to penopubic junction) but is typically isolated to GU anomaly.

Although first characterized in 1709 by Littre and again in 1812 by Meckel [3,4], the prognosis for CE was initially extremely poor with most affected patients dying during infancy secondary to sepsis, gastrointestinal and nutritional issues, and neurologic sequalae [5,6]. The first case of long-term survival following a three-stage operative intervention was reported in 1960 [7]. Due to significant improvements in medical care and surgical reconstructive techniques, survival has since considerably increased, with nearly 100% of patients now surviving [5,8]. Therefore, the focus of modern management has moved to optimizing quality of care and quality of life, including an emphasis on appropriate gender assignment, urinary and fecal social continence, and improved mobility and function [8].

## 2. Epidemiology

Historically, the estimated prevalence of CE was 1 in 200,000 to 400,000 live births [9,10]. More recent studies have suggested higher frequencies [11,12], especially when stillbirths and elective terminations of pregnancy for a fetal anomaly are taken into account (up to 1 in 44,000 births in the country of Wales) [12]. Studies have provided conflicting results on whether CE occurs more frequently in males or females [11,13,14]. This is in contrast to the male predominance witnessed in both bladder exstrophy and epispadias [13]. Potential risk factors for CE have been suggested, including in vitro fertilization and other assisted reproductive techniques [15,16,17], maternal smoking and smoke exposure [14,18], medical radiation [18], and maternal use of clomiphene citrate [19]. CE recurrence within families [20] and increased CE occurrence among conjoined and monozygotic twins [21,22,23,24] has been observed, suggesting that there may be an underlying genetic component [9].

## 3. Embryology and Genetics

Although the cause of CE is not fully understood, several theories on the embryologic origins of CE, and EEC overall, have been proposed. Classically, CE is hypothesized to occur secondary to complications of cloacal membrane development [25,26]. During the fourth week of gestation, overdevelopment of the cloacal membrane prevents medial migration of the mesoderm between the ectoderm and endoderm, leaving the cloacal membrane without mesenchymal support. As a result, the cloacal membrane is unstable and prone to rupture, and normal development of the lower abdominal musculature and pelvic bones are disrupted [1,2]. Notably, the timing and location of cloacal membrane rupture are thought to determine the phenotype along the EEC spectrum. Early rupture of the cloacal membrane prior to fusion of the genital tubercles and descending of the urorectal septum (which eventually divides the gastrointestinal from the genitourinary tracts) results in CE [8]. Conversely, rupture that occurs following urorectal septum development leads to bladder exstrophy or epispadias [2].

However, recent studies and cases have challenged this theory [8,12]. Monozygotic and conjoined twins where only one twin was affected have been reported [23]. Late rupture (i.e., during second trimester) of the cloacal membrane has been observed in several prenatally diagnosed patients with CE [27]. Covered variants of CE have also been described [28,29]. Finally, successful induction of CE in chick embryos suggests an alternate embryologic etiology of CE [30,31].

In recent years, increased attention has been paid to the molecular and genetic etiologies of CE. Reports of recurrence within families [20] and increased CE occurrence among conjoined and monozygotic twins [21,22,23,24] suggest that there is a genetic contribution to CE development. Further support for a genetic etiology is provided by recent identification of copy number variations, susceptibility regions, and genes in CE by array-based, candidate gene association, and genome-wide association studies [9].

## 4. Prenatal Findings and Diagnosis

**Figure 1:**
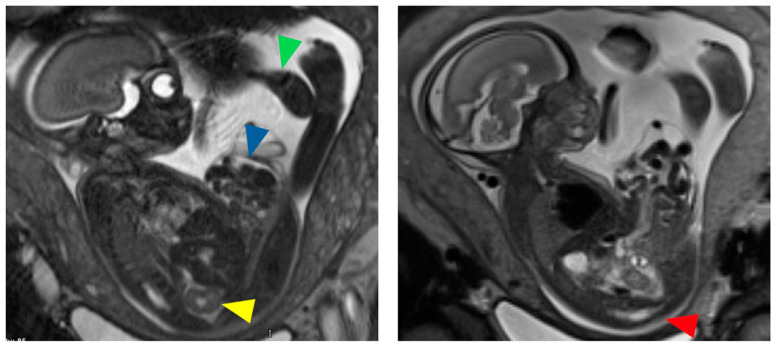
Fetal MRI images of a female fetus at 24 weeks gestation with characteristic findings of cloacal exstrophy including large omphalocele (blue arrow), absent urinary bladder, right pelvic kidney with absent left kidney (yellow arrow), left club foot (green arrow), and low spinal cord termination with truncated sacrum and coccyx (red arrow).

CE can be diagnosed using fetal ultrasonography (US) or magnetic resonance imaging (MRI) [1,32]. Diagnostic criteria for prenatal US were proposed in 1998 based on a multi-institutional study: major criteria (seen in >50% of cases) include non-visualization of the bladder, a large midline infraumbilical anterior wall defect or cystic anterior wall structure (i.e., persistent cloacal membrane), omphalocele, and lumbosacral anomalies [33] (Figure 1). Additionally, minor criteria (seen in <50% of cases) were defined to include lower extremity defects, renal anomalies, ascites, widened pubic arches, a narrow thorax, hydrocephalus, and a single umbilical artery [33]. The prolapsed ileum associated with CE may appear like an ‘elephant trunk’ on prenatal US [34]. Until recently, rates of prenatal diagnosis of CE were reported to be ~50% despite these characteristic US findings [14]. This may, in part, stem from confusion with isolated omphalocele, bladder exstrophy, and other midline abdominal wall defects [14,35]. A recent single institutional registry study determined that identifying the location of umbilical cord insertion relative to the abdominal wall defect, either by fetal US or MRI, is critical to accurately differentiating between CE and bladder exstrophy (inferior insertion suggests CE) [35].

Two recent studies suggest that current rates of prenatal diagnosis of CE are considerably higher than previously reported. Review of a multi-institutional database demonstrated a prenatal diagnosis rate of 82.2% for CE between 2000 and 2020 [32]. A large single institution study found a similar prenatal diagnosis rate (78.6%) for this time period [36]; it was further noted that rates have increased from 56.3% during 2000–2006 to 88.9% during 2014–2020 [36]. An accurate prenatal diagnosis permits education, counseling, and preparation of expectant parents [37]. Additionally, it provides time to plan and coordinate a safe pregnancy, timing and location of delivery, optimal neonatal management, and operative planning [1,8,36]. Following prenatal diagnosis, patients may be most appropriately taken care of by an experienced multidisciplinary team, especially as earlier presentation to such centers has been shown to impact rates of successful closure [36].

## 5. Anatomy and Associated Anomalies

**Figure 2:**
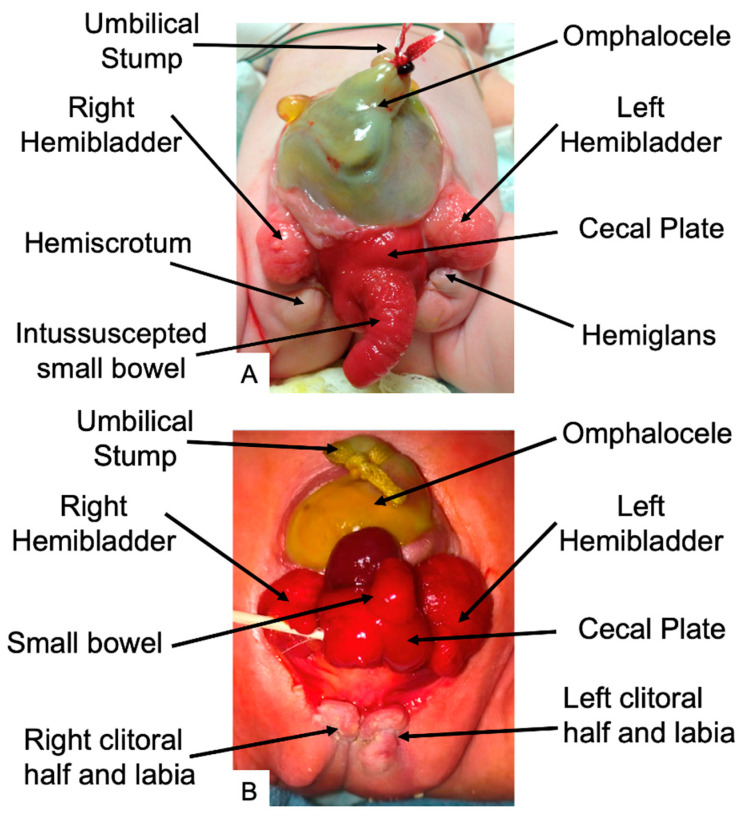
Phenotypic characteristics of cloacal exstrophy in a (**A**) male infant and (**B**) female infant (Figure 2A adapted from [1]).

The classic anatomic presentation of CE includes an omphalocele of varying size, exstrophy of two small hemibladders with a central exstrophic cecum, imperforate anus, and anomalous genitalia [38,39] (Figure 2). The exstrophic cecum is frequently accompanied by a prolapse of the terminal ileum, leading to the appearance of an elephant’s trunk deformity [8]. Anomalies outside of the classic CE complex, frequently involving the gastrointestinal tract, genitourinary system, spine, and musculoskeletal system, occur in 90% of patients [40] and can be the source of significant morbidity [1]. Preterm delivery is not uncommon with a recent single institution review reporting delivery at a median of 33 weeks for their CE population [41]. The sequalae of premature delivery, as well as pulmonary hypoplasia in some patients with large omphaloceles, can lead to additional morbidity and occasionally mortality [41,42].

### 5.1. Gastrointestinal Anomalies

Associated anomalies of the gastrointestinal tract can be a source of significant morbidity and mortality [43]. Congenital short bowel syndrome has been observed in up to 25% of patients, underscoring the importance of ileal and colonic preservation [8,44]. Other findings include intestinal duplications (including of the appendix, ileum, and colon), gastroschisis, ectopic perineal anus, malrotation, and duodenal atresia [40,43,44].

### 5.2. Genitourinary Anomalies

Genitourinary anomalies are estimated to occur in 42–60% of patients with CE [8,40]. Abnormalities of the kidneys are common (48% of patients), including unilateral renal agenesis, renal ectopia, malrotation, collecting system duplication, congenital cysts, and ureterovesical junction obstruction [40,45]. Ureteral atresia and bladder duplication have also been described [8,40]. Functional renal impairment can also be present, though this appears to be related to postnatal injury as baseline renal volumes are similar between infants with and without CE [8,46]. Children with solitary kidneys require close follow-up and renal protection. Following urinary reconstruction, the upper tracts are at risk of damage from high pressure bladder dynamics, pyelonephritis, and vesicoureteral reflux. Vesicoureteral reflux has been reported in 50 to 60% following reconstruction and may need to be addressed at time of urinary reconstruction [47]. The prevalence of pelvic ectopic kidney is 16–30%. Acute renal dysfunction in pelvic ectopic kidneys are of particular concern during bladder closure due to an abrupt increase in abdominal/pelvic pressure following pubic bone approximation during second-stage OEIS closure (e.g., compartment syndrome) [48].

In boys, the phallus is frequently small and bifid with wide division of the scrotum [6,49]. Intravesical location of the phallus (which may be mistaken as aphallia) has also been described [50,51]. Historically, gender reassignment was often undertaken in the setting of a diminutive phallic structure, which frequently led to significant psychological impacts [49]. Undescended testicles are common, and anomalies of the vas deferens have been described, including absence and duplication [8]. The practice of gender reassignment has largely been abandoned, and there have been advancements in reconstruction.

In girls, separated clitoral halves and wide division of the labia are typical [6,45]. Further, Müllerian anomalies are very common, occurring in 87% of patients with CE [45]. Uterus didelphys is the most frequently encountered anomalies (70% in a recent study) [45,52]. It is usually associated with vaginal duplication. Vaginal anomalies include duplication, agenesis, atresia/hypoplasia, and lateral displacement [52]. Cervical duplication [52] and rarely ovarian duplication [40] may also occur.

### 5.3. Neurospinal Anomalies

Complex spinal dysraphisms are present in nearly all patients with CE with two recent institutional studies showing rates of 97–98% [53,54]. Lipoma-based defects are the most common [53,54]. The occurrence of myelocystocele, myelomeningocele, low-lying conus with tethered cord/fatty filum, diastematomyelia, and meningocele have also been described [53,54]. Given the high rates of spinal dysraphism in this population, screening is highly recommended with any abnormal findings prompting neurosurgical evaluation. Additionally, nearly a third of patients have been found to have an intracranial abnormality, including hydrocephalus, Chiari malformation, and craniosynostosis [55]. Patients with a spinal dysraphism are at particular risk for a concomitant intracranial anomaly [53].

### 5.4. Musculoskeletal Anomalies

CE is associated with anomalies in the pelvis, vertebral column, and extremities. The pelvis in CE has a large diastasis between the pubic rami, sometimes upwards of 6 cm, as well as external angling of the anterior and posterior segments and external rotation and abduction of the iliac wings [6,56]. Frequently, there is shortening of bone in the anterior pelvis by over 40% [56]. Hip dysplasia has been found in 16% of patients and should thus prompt physical examination and plain radiographs in the CE population [57]. Vertebral anomalies may include scoliosis, kyphosis, hemivertebrae, sacral abnormalities, and vertebral duplication [40]. Lower limb orthopedic malformations include club foot, equinovarus deformity, and various anomalies of the digits [6,8].

### 5.5. Differential Diagnosis

Particularly in the prenatal setting, an accurate diagnosis of CE can be challenging. The anterior abdominal wall defect, most commonly an omphalocele, may be noted, but additional imaging findings concerning for CE may not be recognized [36]. Using prenatal imaging alone, the differential diagnosis may include an isolated omphalocele, bladder exstrophy, and covered CE. Using both prenatal US and fetal MRI, bladder exstrophy can be misdiagnosed as CE due to visualization and misinterpretation of the everting bladder plate with bowel loops posterior to the plate [35]. Recognizing location of umbilical cord insertion relative to the abdominal wall defect is critical in differentiating between bladder and cloacal exstrophy prenatally [35]. Postnatal physical examination findings can typically lead to the correct diagnosis, with the exception of covered CE variants, which can be exceptionally difficult to diagnose [58]. The presence of the characteristic set of malformations in CE (omphalocele, two hemibladders with exstrophic cecum, imperforate anus) permits differentiation from an isolated omphalocele and bladder exstrophy.

## 6. Postnatal Evaluation and Management

**Figure 3:**
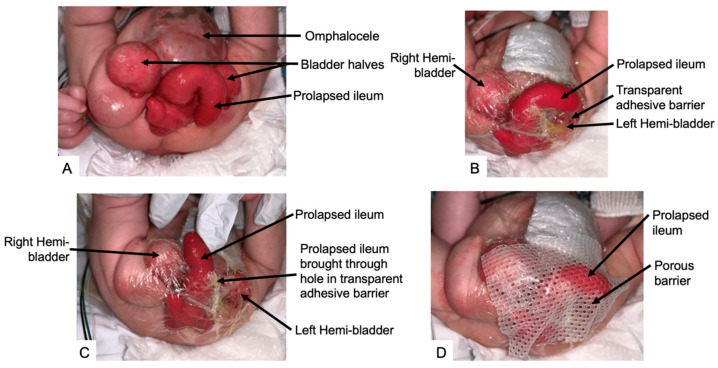
Coverage of bladder and cecal plate. (**A**) Anatomy of cloacal exstrophy; (**B**) application of transparent adhesive barrier to protect bladder halves and cecal plate and to separate them from the stool emanating from the prolapsed ileum; (**C**) prolapsed ileum is brought through a hole in the transparent adhesive barrier; (**D**) application of porous barrier to protect the prolapsed ileum from diaper abrasion.

Immediately following birth, stabilization of the newborn, coverage of the exposed omphalocele, bladder, and bowel, and protection of the myelomeningocele should be prioritized. Options for coverage of the bladder and cecal plate mucosa include an occlusive plastic wrap or a hydrated silicone gel dressing (Figure 3). This aids in preventing abrasions from the diaper, keeping bladder plates moist, and decreasing infection risk [6,59]. The umbilical cord should be ligated with suture rather than an umbilical clamp as this can abrade the bladder, cecal plate, and prolapsed ileum [59,60]. Nutrition should be optimized to facilitate growth and development and postoperative wound healing.

A detailed physical examination should assess for associated anomalies and determine the presence of a phallus or vaginal tissue. Baseline laboratory studies, including hematologic status, renal function and serum electrolytes, and blood typing, should be obtained. Karyotyping should be considered if chromosomal sex is not clear during examination [60]. Initial evaluation should include a spinal US to assess for spinal dysraphism; spinal MRI may be advisable if lesions are unclear or anatomy needs to be further defined [59]. Additionally, anterior–posterior radiography of the pelvis provides information about distance between pubic rami [59]. Renal US should be performed for evaluation of the upper urinary tract. Multidisciplinary assessment and management of these infants should be undertaken and may involve pediatric urologists, pediatric surgeons, pediatric gynecologists, orthopedic surgeons, neurosurgeons, gastroenterologists, endocrinologists, nephrologists, and neonatologists depending on the associated anomalies identified [60].

## 7. Operative Management

Given the spectrum of anatomy, physiology, and associated anomalies seen in CE, each patient necessitates a personalized approach to their operative repair. Generally, this will include omphalocele repair, separation of the hindgut segment with colostomy, and bladder closure, which often necessitates the approximation of the pubic diastasis with osteotomy [60,61]. Several approaches and techniques have been proposed, including a one-stage approach versus a staged approach, potential need for osteotomy, and type of osteotomy. Separation of the urological and gastrointestinal systems within 48–72 h after birth has traditionally been recommended to minimize risk of omphalocele rupture, urinary tract infection, and metabolic issues secondary to colonic mucosa being exposed to urine [41]. The timing of the initial intervention is highly dependent on the stability of the patient as well as their associated anomalies. In particular, premature patients and those with pulmonary issues related to prematurity or pulmonary hypoplasia may need delay of the first stage of reconstruction. Delay of the separation of the bladder and cecal plate beyond the immediate postnatal period in these settings has been shown to be safe without increased risk of infection or metabolic complications but may have longer lengths of stay and use of parental nutrition while admitted [41].

If delay of repair is needed, special attention to the omphalocele and exstrophy plate is required. Dressings can be fashioned to protect the bladder plate and separate stool (Figure 3). In cases with an open spinal dysraphism or large myelocystocele, closure may be warranted prior to repairing the abdominal defect. Discussions with pediatric surgery, urology, and neurosurgery teams should prioritize the sequence of surgeries. Additionally, severe congenital cardiac anomalies may require intervention before the abdomen can be safely reconstructed.

With survival no longer being the main concern for most patients with CE, the focus has been able to shift to outcomes and quality of life. Surgical techniques and approaches have evolved with this in mind [39,47,62,63,64]. Many teams have moved from single-stage reconstruction to a staged approach with single-stage reconstruction only considered in rare cases with optimal anatomy [60,65]. A staged approach is safe and successful [66] with the odds of a successful primary bladder closure being four times greater for the staged approach than the single-stage approach [61]. The remainder of this review will focus on the modern approaches and techniques taken in the staged reconstructive approach.

### 7.1. Stage 1 Closure (Newborn)

The steps of the first stage of reconstruction include (1) separation of the cecal plate, (2) rescue of the hindgut, (3) tubularization of the cecum, (4) reapproximation of the bladders halves, (5) possible omphalocele closure versus delayed closure, and (6) creation of an end colostomy [1,60].

At the time of initial repair, a full genitourinary examination under anesthesia should first be performed to confirm anatomy. The ureteral orifices are then cannulated with 3.0–3.5 Fr ureteral catheters, which are secured in place. Dissection of the omphalocele is started inferiorly and separated from the bladder plates [8,60]. The umbilical vessels are ligated, followed by separation of the bladder plates from adjacent skin.

#### 7.1.1. Bowel Reconstruction

Typically, the medial cecal plate is then separated from the two lateral hemi-bladders. There is ongoing debate on whether to retain all or a portion of the cecal plate in the bladder closure for augmentation purposes versus keeping the cecal plate in the gastrointestinal tract to maximize bowel length and water absorption [60]. To incorporate the cecal plate into the gastrointestinal tract following separation from the hemibladders, the cecal plate is tubularized to create continuity from the terminal ileum via the cecum to the blind-ending hindgut. Following mobilization of the blind-ending hindgut, an end colostomy is matured with care given to optimal colostomy placement based on the patient’s individual anatomy and reconstructive needs [8].

Historically, some patients underwent the creation of a terminal ileostomy at birth, leaving the colon defunctionalized and in situ [60,67]. Analysis of patients with an ileostomy compared to those with a colostomy showed an increased length of stay secondary to gastrointestinal complications and increased days on supplemental parenteral nutrition [68]. A ‘rescue operation’ (involving rescue of the colon from the pelvis, creation of gastrointestinal continuity, closure of ileostomy, and creation of colostomy) is now the more standard approach to allow for the entire GI tract to be in continuity [67]. This operation is able to achieve resolution of pre-operative symptoms that occur secondary to the persistent connection between the genitourinary tract and the hindgut, including hyperchloremic acidosis, urinary tract infections, failure to thrive, sepsis, dehydration, and TPN dependence [67].

Intestinal duplications (ileum, colon, and/or appendix) are present in some patients. No intestinal segments, including the appendix, should be sacrificed as they can frequently be utilized during reconstruction of bowel, bladder and/or vagina [8,60]. If present, the appendix may be used to construct a catheterizable channel to promote urinary continence, which is particularly valuable given that urinary continence is most frequently achieved by clean channel catheterization in this population [69]. Small and large bowel segments can be used to construct a neovagina [52,70] and for urinary tract augmentation [71]. Duplicated colonic segments may also be unified in sequence or the wall removed in between to prolong intestinal transit time and prevent stool stasis [8,72].

#### 7.1.2. Bladder Reconstruction

The two lateral hemibladders are then joined in the midline to create a single posterior bladder plate. Care must be taken to protect the ureteral orifices. This essentially converts a CE into bladder exstrophy [1]. The exposed bladder mucosa should be protected with an occlusive dressing, similar to the preoperative dressing (Figure 3). In a single-stage procedure, bladder reconstruction would continue with anterior closure to form a closed bladder that is placed behind the approximated pubic rami [8,66].

#### 7.1.3. Omphalocele Closure

Omphalocele closure depends on the size and the ability to safely reduce the contents with adequate abdominal domain without causing morbidity. Closure in the first stage is possible if there is no evidence of abdominal compartment syndrome and respiratory compromise. Peak inspiratory pressures and tidal volumes are re-monitored intra-operatively [59]. A staged closure may be necessary, and a silo or biosynthetic patch for bridging of the fascial defect can be employed [60].

### 7.2. Stage 2 Closure (6 Months–2 Years of Age)

The goals of the second stage of reconstruction include (1) bladder closure, (2) abdominal wall closure, and (3) reconstruction of external genitalia in select patients. Inguinal hernias should be repaired if found. This is typically performed between 6 months and 2 years of age but depends on the nutritional status and growth of the patient [1,8]. Pubic bone approximation leads to increased abdominal and pelvic pressure, which may be of particular concern in patients with a pelvic ectopic kidney. During closure, pelvic ectopic kidneys have been shown to incur a more significant increase in renal pelvis pressure, peak systolic velocity, and resistive index compared to orthotopic kidneys [48]. Real-time monitoring of renal pelvis pressure in the perioperative period should therefore be considered in patients with a pelvic ectopic kidney, especially in those with a solitary kidney [48]. Additional potential complications of this stage include bladder dehiscence, penile ischemia, urethrocutaneous or vesicocutaneous fistula, bladder outlet obstruction, urinary tract infection, compromised renal function, and prolonged ileus [1].

Pelvic osteotomy is frequently recommended as part of closure to decrease abdominal wall tension following pubic bone approximation [61,65]. It has also been shown that an osteotomy significantly increases the chance of a successful bladder closure and decreases risk of wound complications [61,73,74]. Various types of pelvic osteotomy have been proposed, including bilateral posterior iliac, bilateral transverse anterior innominate, or combined bilateral anterior innominate and vertical iliac osteotomy, and the preference of osteotomy type varies by center [61,65]. Osteotomy can be performed on the day of bladder reconstruction or as a staged procedure with osteotomy occurring 2–3 weeks prior to bladder closure [56]. It has been shown that by decreasing a very wide public bone diastasis (>6.0 cm), increased rates of successful closure are achieved without an increase in complications with the use of a staged osteotomy [65,75]. Various post-operative immobilization techniques have been described, including Bryant’s traction, spica casting, Buck’s traction, and external fixation [61].

### 7.3. Additional Interventions

Many patients with CE will require secondary reconstructive procedures at a later age involving their urinary and gastrointestinal tracts, often in attempts to achieve urinary and fecal continence. Additional operations may also be performed for genital reconstruction but must take into consideration gender assignment and identity. These secondary interventions must be individualized to the patient, their anatomy, and goals.

Secondary to spinal dysraphism frequently found with CE, many patients will not develop continence with volitional voiding [71]. Urinary reconstruction must be individualized to meet their needs, as many can achieve social continence through urologic reconstruction and, for some patients, the use of a catheterizable channel [5]. Therefore, a “third stage” of reconstruction most commonly includes bladder neck closure, bladder augmentation to increase bladder capacity, possible correction of vesicoureteral reflux, and the creation of a catheterizable channel for timed bladder emptying [59]. Bladder augmentation can be performed using the stomach, ileum and/or colon [76,77]. Patients and parents/caregivers must be socially ready for the “third stage” of reconstruction, as nonadherence to clean intermittent catheterization of the catheterizable channel can result in complications such as bladder perforation or renal injury from reflux and elevated upper tract pressure (related to high pressures in the bladder from not emptying).

A small number of patients with CE may undergo colonic pull-through [5,78,79]. Due to the overall low rate of fecal continence in this population, controversy exists regarding performing these procedures in patients with CE. However, careful patient selection and commitment to bowel management may reveal a subset of patients that can benefit [79]. Recently, colon length has been shown to correlate with a successful outcome following pull-through: clean patients had an average of 64.0 cm of colon compared to those who are not clean or opt for a re-do ostomy (26.5 cm) [80].

## 8. Gender Assignment and Identity

Gender assignment for karyotypically male patients with CE continues to be a topic of active discussion and controversy. Many genetically male infants have very diminutive phallic structures that cannot be easily reconstructed. Therefore, the majority of these infants were historically surgically gender reassigned during infancy and raised as female [2,8,40]. However, improved recognition of the complexities of gender identity and increased focus on quality of life have led to changes in this practice. Prenatal androgen exposure and androgenization (i.e., androgen imprinting) in karyotypically male neonates may predispose to a male gender identity [81,82,83]. In the early 2000s, an important study demonstrated that 8 of 14 genetically karyotypically male patients re-assigned to the female gender in infancy had self-reconverted to the male gender during adolescence and adulthood [84]. Further, reassigned patients were found to have significantly higher rates of depression than non-assigned patients [85].

Subsequently, there has been a movement towards avoiding gender re-assignment in infancy and raising karyotypically male infants as males [8]. A single institution retrospective study captures this trend: between 1985 and 1992, 54% underwent sex conversion compared to 6% between 2001 and 2008 [40]. Surveys of American pediatric urologists performed in 2004 and again in 2010 showed an increasing belief in male gender assignment for karyotypically male patients with CE (70% to 79%) [86,87]. Neophallus creation utilizing microvascular techniques may be indicated in the most severe cases [60].

## 9. Long-Term Outcomes

Following improvements in neonatal care and surgical reconstructive techniques, patients with CE are living longer lives. However, there is a paucity of data on long-term outcomes and quality of life [88]. Further, there is a lack of standardized definitions across studies, especially in defining continence, making comparisons challenging [89].

### 9.1. Urinary Outcomes

Given frequent spinal anomalies and complex anatomical malformations in CE, volitional urinary control and detrusor contractions are infrequently possible [1]. A recent large multi-institutional study of 160 patients, including children, older children, and adults, found that 42% of children under the age of 10 years were incontinent and wearing diapers and 32% were on clean intermittent catheterization (CIC) [90]. Among older children (age 10 to 18 years) and adults, 73% were on CIC with 88% having a history of bladder augmentation and 89% utilizing a catheterizable channel. In adults, incontinent urinary diversion was not uncommon, occurring in 28%. Of note, in this series, no child or adult voided spontaneously per urethra [90]. In a recent single-institution study of long-term (>10 years) outcomes of patients with CE (*n* = 63), most patients (71.4%) had a catheterizable stoma, and of these patients, 88.9% were considered continent between catheterizations [5]. Ileal conduits were found in 9.5% of patients. Notably, only three patients (4.8%) were catheterized per urethra with only one of these patients achieving continence [5]. Several smaller studies provide data on urinary continence; however, it is difficult to interpret secondary to a small study population and variable definitions [76,85,91]. Although urinary continence due to CIC is an achievable goal for many patients with CE, considerable commitment, time, and financial burden is required [69,92]. In a series of 116 patients with CE, the median number of urologic procedures needed to achieve urinary continence was 4 (range 2–10) with a median time of 11 years to urinary continence [69].

### 9.2. Gastrointestinal Outcomes

Studies describing long-term gastrointestinal outcomes, including fecal continence, are very limited. A recent multi-institutional study found that 79% of patients had an intestinal diversion, regardless of birth year [90]. The remaining patients underwent a pull-through with 50% additionally undergoing a Malone antegrade colonic enema (MACE) procedure. Notably, rates of intestinal diversion varied significantly across centers from 55% to 91% [90]. A recent study showed similarly high rates of intestinal diversion (92.1%) with permanent colostomy being the most common (61.9%), followed by ileostomy (30.2%). In this series, 7.9% of patients underwent a PSARP and three of these five patients were able to achieve fecal continence (only one patient remains on a bowel regimen) [5]. Published rates of continence following pull-through vary widely (rates of being clean: 30–85%), and continued debate about whether this should be attempted persists [79,80,90].

### 9.3. Renal Outcomes

Patients with CE have a baseline elevated risk of chronic kidney disease given the high frequency of underlying renal anomalies, including agenesis, dysplasia, fusion, and ectopia [1]. Vesicoureteral reflux, urinary tract infections/pyelonephritis, and stones are well-described in this population and lead to kidney damage [77]. Additionally, kidneys are at risk of acute dysfunction during abdominal closure in stages 1 and 2 of reconstruction [48]. Most available studies focus on subsets of patients with CE (i.e., those who have undergone certain procedures), so it is difficult to ascertain the risk of renal dysfunction in the overall CE population. However, a recent single-institution study supports that there is a considerable risk of chronic kidney disease, with 25% of their CE population having at least stage 1 chronic kidney disease [5].

### 9.4. Growth and Ambulation Outcomes

The complex anatomic malformations associated with CE, as well as the physiologic derangements that result from these malformations and from reconstructive procedures, may portend a risk of growth morbidity. Patients with CE have been shown to have significantly lower median height-for-age and weight-for-age z-scores than the general population [93]. In this study, short bowel syndrome and enterocystoplasty with the intestine were found to be associated with lower z-scores [93]. Nutritional optimization may be able to, at least partially, mitigate these growth issues. This should be instituted as a neonate and carried throughout childhood with enteral and parental nutrition as needed. Bone mass appears to be preserved when corrected for small stature [94].

Few studies comment on ambulation and mobility, despite the high prevalence of spinal anomalies and lower extremity malformations in this population [5,78,84,85]. The largest study providing relevant data (*n* = 63) reports ambulation without aids in 36.5%, wheelchair dependence in 34.9%, abnormal gait in 12.7%, a need for leg braces or a walker adjunct in 14.3%, and being fully immobilized in 1.6% [5].

### 9.5. Sexual Function and Fertility

Sexual function and fertility is likely to be impacted in patients with CE given the high prevalence of severe genital anomalies and complex Müllerian abnormalities [52]. However, the available data are highly limited given that CE has become a survivable malformation only relatively recently.

Considering the female population, a recent systematic review suggested that only 17.9% reported being sexually active across four studies [78]. Successful conception, pregnancy, and delivery have been reported in case reports and case series [5,52,95,96,97,98]. One patient with CE has carried three pregnancies, and there are documentations of pregnancy loss, uterine prolapse, preterm birth, and cesarean birth [5,52,95,96,97,98]. However, the number of pregnancies in patients with CE is too small to quantify and provide meaningful analysis of fertility, pregnancy complications, and mode of delivery.

Data for the male population are even more limited, in part due to the historical decision to raise genetically male patients with a diminutive phallus not amenable for reconstruction as female [1]. However, testicular histology appears to be preserved, and no significant abnormalities in the architecture of the rete testis, epididymis or vas are found in genotypic male patients with CE [49]. No case of natural paternity in a genotypic male patient with CE has been documented in the literature [78].

### 9.6. Psychosocial Outcomes

Psychosocial outcomes in patients with CE are also poorly studied with only one study from the Hopkins group providing relevant but concerning data [5]. Anxiety and/or depression were noted in two-thirds of their patients, with chronic pain present in over one-fourth of patients. Social outcomes were more promising, with it being noted that nearly 80% of adult patients had attended college and over 80% were employed [5].

## 10. Discussion

In recent years, the care of patients with CE has advanced considerably. Cloacal exstrophy has evolved from being a universally fatal malformation prior to the 1960s to now attaining the survival of nearly all patients with this condition. With survival no longer having to be the primary concern, the emphasis has shifted to achieving improved long-term quality of life. Modifications are continuously being made to the operative approach with this goal in mind. Additionally, gender reassignment of karyotypically male patients with CE, once a common practice, is no longer standard practice.

Multidisciplinary teams, including pediatric urology, pediatric colorectal surgery, orthopedic surgery, gastroenterology, and neurosurgery, have been established within specialized centers at many institutions. Improvements in neonatal care and understanding of the physiology of these patients has afforded the ability to safely delay reconstructive surgery [41]. This permits the optimization of severe medical co-morbidities and associated anomalies, as well as hydration and nutrition prior to major operative intervention. It is now well-accepted that staged reconstruction may achieve improved long-term outcomes. Following the recognition of significant multifactorial long-term growth failure in patients with CE, increased attention is also being given to nutrition and growth [93].

Due to its rarity, complexity, and the highly individualized anatomy of patients with CE, long-term outcome data on function and quality of life are lacking. Currently, most of the studies on CE result from single-institution studies. To further improve the management of CE and provide families with realistic expectations, multi-institutional study consortiums are needed.

## 11. Conclusions

Cloacal exstrophy remains a challenging congenital anomaly affecting multiple organ systems. Advances in medical care and surgical reconstructive techniques have led to near-universal survival. Subsequently, optimizing quality of care and quality of life has come into focus, including an emphasis on gender identity, urinary and fecal continence, and improved mobility and function. An individualized approach to each patient by an experienced multidisciplinary team is essential for achieving the best outcomes.

## Data Availability

No new data were created or analyzed in this study. Data sharing is not applicable to this article.

## References

[1] Lee T., Borer J. (2023). Exstrophy-Epispadias Complex. Urol. Clin. N. Am..

[2] Inouye B.M., Tourchi A., Di Carlo H.N., Young E.E., Gearhart J.P. (2014). Modern Management of the Exstrophy-Epispadias Complex. Surg. Res. Pract..

[3] Littre A. (1709). Diverse Observations Anatomique. Mem. Acad. Roy. Sc..

[4] Meckel J.F. (1812). Handbuch Der Pathologischen Anatomie.

[5] Haney N.M., Morrill C.C., Haffar A., Crigger C., Gabrielson A.T., Galansky L., Gearhart J.P. (2024). Long-Term Management of Problems in Cloacal Exstrophy: A Single-Institution Review. J. Pediatr. Surg..

[6] Phillips T.M. (2011). Spectrum of Cloacal Exstrophy. Semin. Pediatr. Surg..

[7] Rickham P.P. (1960). Vesico-Intestinal Fissure. Arch. Dis. Child..

[8] Wilcox D., Bischoff A., Ziegler M.M. (2023). Cloacal Exstrophy. Pediatric Surgery Diagnosis and Management.

[9] Reutter H., Keppler-Noreuil K., E Keegan C., Thiele H., Yamada G., Ludwig M. (2016). Genetics of Bladder-Exstrophy-Epispadias Complex (BEEC): Systematic Elucidation of Mendelian and Multifactorial Phenotypes. Curr. Genom..

[10] Martínez-Frías M.L., Bermejo E., Rodríguez-Pinilla E., Frías J.L. (2001). Exstrophy of the Cloaca and Exstrophy of the Bladder: Two Different Expressions of a Primary Developmental Field Defect. Am. J. Med. Genet..

[11] Caton A.R., Bloom A., Druschel C.M., Kirby R.S. (2007). Epidemiology of Bladder and Cloacal Exstrophies in New York State, 1983–1999. Birth Defects Res. Part A Clin. Mol. Teratol..

[12] Feldkamp M.L., Botto L.D., Amar E., Bakker M.K., Bermejo-Sánchez E., Bianca S., Canfield M.A., Castilla E.E., Clementi M., Csaky-Szunyogh M. (2011). Cloacal Exstrophy: An Epidemiologic Study from the International Clearinghouse for Birth Defects Surveillance and Research. Am. J. Med. Genet. C Semin. Med. Genet..

[13] Boyadjiev S.A., Dodson J.L., Radford C.L., Ashrafi G.H., Beaty T.H., Mathews R.I., Broman K.W., Gearhart J.P. (2004). Clinical and Molecular Characterization of the Bladder Exstrophy-Epispadias Complex: Analysis of 232 Families. BJU Int..

[14] Gambhir L., Höller T., Müller M., Schott G., Vogt H., Detlefsen B., Ebert A.-K., Fisch M., Beaudoin S., Stein R. (2008). Epidemiological Survey of 214 Families With Bladder Exstrophy-Epispadias Complex. J. Urol..

[15] Wood H.M., Babineau D., Gearhart J.P. (2007). In Vitro Fertilization and the Cloacal/Bladder Exstrophy-Epispadias Complex: A Continuing Association. J. Pediatr. Urol..

[16] Wood H.M., Trock B.J., Gearhart J.P. (2003). In Vitro Fertilization and the Cloacal-Bladder Exstrophy-Epispadias Complex: Is There an Association?. J. Urol..

[17] Zwink N., Jenetzky E., Hirsch K., Reifferscheid P., Schmiedeke E., Schmidt D., Reckin S., Obermayr F., Boemers T.M., Stein R. (2013). Assisted Reproductive Techniques and Risk of Exstrophy-Epispadias Complex: A German Case-Control Study. J. Urol..

[18] Reutter H., Boyadjiev S.A., Gambhir L., Ebert A.-K., Rösch W.H., Stein R., Schröder A., Boemers T.M., Bartels E., Vogt H. (2011). Phenotype Severity in the Bladder Exstrophy-Epispadias Complex: Analysis of Genetic and Nongenetic Contributing Factors in 441 Families from North America and Europe. J. Pediatr..

[19] Reefhuis J., Honein M.A., Schieve L.A., Rasmussen S.A. (2011). National Birth Defects Prevention Study Use of Clomiphene Citrate and Birth Defects, National Birth Defects Prevention Study, 1997–2005. Hum. Reprod..

[20] Smith N.M., Chambers H.M., Furness M.E., Haan E.A. (1992). The OEIS Complex (Omphalocele-Exstrophy-Imperforate Anus-Spinal Defects): Recurrence in Sibs. J. Med. Genet..

[21] Fullerton B.S., Sparks E.A., Hall A.M., Velazco C.S., Modi B.P., Lund D.P., Jaksic T., Hendren W.H. (2017). High Prevalence of Same-Sex Twins in Patients with Cloacal Exstrophy: Support for Embryological Association with Monozygotic Twinning. J. Pediatr. Surg..

[22] Lee D.H., Cottrell J.R., Sanders R.C., Meyers C.M., Wulfsberg E.A., Sun C.C. (1999). OEIS Complex (Omphalocele-Exstrophy-Imperforate Anus-Spinal Defects) in Monozygotic Twins. Am. J. Med. Genet..

[23] Casale P., Grady R.W., Waldhausen J.H.T., Joyner B.D., Wright J., Mitchell M.E. (2004). Cloacal Exstrophy Variants. Can Blighted Conjoined Twinning Play a Role?. J. Urol..

[24] Siebert J.R., Rutledge J.C., Kapur R.P. (2005). Association of Cloacal Anomalies, Caudal Duplication, and Twinning. Pediatr. Dev. Pathol..

[25] Gray S.K., Skandalakis J. (1972). The Colon and Rectum. Embryology for Surgeons.

[26] Marshall V.F., Muecke E.C. (1962). Variations in Exstrophy of the Bladder. J. Urol..

[27] Bruch S.W., Adzick N.S., Goldstein R.B., Harrison M.R. (1996). Challenging the Embryogenesis of Cloacal Exstrophy. J. Pediatr. Surg..

[28] Sahoo S.P., Gangopadhyay A.N., Sinha C.K., Gupta D.K., Gopal S.C. (1997). Covered Exstrophy: A Rare Variant of Classical Bladder Exstrophy. Scand. J. Urol. Nephrol..

[29] Borwankar S.S., Kasat L.S., Naregal A., Jain M., Bajaj R. (1998). Covered Exstrophy: A Rare Variant. Pediatr. Surg. Int..

[30] Männer J., Kluth D. (2005). The Morphogenesis of the Exstrophy-Epispadias Complex: A New Concept Based on Observations Made in Early Embryonic Cases of Cloacal Exstrophy. Anat. Embryol..

[31] Männer J., Kluth D. (2003). A Chicken Model to Study the Embryology of Cloacal Exstrophy. J. Pediatr. Surg..

[32] Lee T., Weiss D., Roth E., Bortnick E., Jarosz S., Eftekharzadeh S., Groth T., Shukla A., Kryger J.V., Lee R.S. (2023). Prenatal Diagnosis of Bladder Exstrophy and OEIS over 20 Years. Urology.

[33] Austin P.F., Homsy Y.L., Gearhart J.P., Porter K., Guidi C., Madsen K., Maizels M. (1998). The Prenatal Diagnosis of Cloacal Exstrophy. J. Urol..

[34] Hamada H., Takano K., Shiina H., Sakai T., Sohda S., Kubo T. (1999). New Ultrasonographic Criterion for the Prenatal Diagnosis of Cloacal Exstrophy: Elephant Trunk-like Image. J. Urol..

[35] Weiss D.A., Oliver E.R., Borer J.G., Kryger J.V., Roth E.B., Groth T.W., Shukla A.R., Mitchell M.E., Canning D.A., Victoria T. (2020). Key Anatomic Findings on Fetal Ultrasound and MRI in the Prenatal Diagnosis of Bladder and Cloacal Exstrophy. J. Pediatr. Urol..

[36] Morrill C.C., Haffar A., Crigger C., Black M., Jelin A., Nasr I., Gearhart J.P. (2023). A Single-Institutional Experience With Prenatal Diagnosis of Cloacal Exstrophy: Room for Improvement. J. Pediatr. Surg..

[37] Bischoff A., Calvo-Garcia M.A., Baregamian N., Levitt M.A., Lim F.-Y., Hall J., Peña A. (2012). Prenatal Counseling for Cloaca and Cloacal Exstrophy-Challenges Faced by Pediatric Surgeons. Pediatr. Surg. Int..

[38] Woo L.L., Thomas J.C., Brock J.W. (2010). Cloacal Exstrophy: A Comprehensive Review of an Uncommon Problem. J. Pediatr. Urol..

[39] Lund D.P., Hendren W.H. (2001). Cloacal Exstrophy: A 25-Year Experience with 50 Cases. J. Pediatr. Surg..

[40] Sawaya D., Goldstein S., Seetharamaiah R., Suson K., Nabaweesi R., Colombani P., Gearhart J. (2010). Gastrointestinal Ramifications of the Cloacal Exstrophy Complex: A 44-Year Experience. J. Pediatr. Surg..

[41] Tirrell T.F., Demehri F.R., Henry O.S., Cullen L., Lillehei C.W., Warf B.C., Gates R.L., Borer J.G., Dickie B.H. (2021). Safety of Delayed Surgical Repair of Omphalocele-Exstrophy-Imperforate Anus-Spinal Defects (OEIS) Complex in Infants with Significant Comorbidities. Pediatr. Surg. Int..

[42] Argyle J.C. (1989). Pulmonary Hypoplasia in Infants with Giant Abdominal Wall Defects. Pediatr. Pathol..

[43] McHoney M., Ransley P.G., Duffy P., Wilcox D.T., Spitz L. (2004). Cloacal Exstrophy: Morbidity Associated with Abnormalities of the Gastrointestinal Tract and Spine. J. Pediatr. Surg..

[44] Davidoff A.M., Hebra A., Balmer D., Templeton J.M., Schnaufer L. (1996). Management of the Gastrointestinal Tract and Nutrition in Patients with Cloacal Exstrophy. J. Pediatr. Surg..

[45] Suson K.D., Inouye B., Carl A., Gearhart J.P. (2016). Congenital Renal Anomalies in Cloacal Exstrophy: Is There a Difference?. J. Pediatr. Urol..

[46] Rehfuss A., Bourgeois T., Thompson B., Sebastião Y.V., Wood R.J., Jayanthi V.R., Fuchs M.E. (2021). Baseline Renal Volumes in Children Born With Cloacal Anomalies. Urology.

[47] Stolar C.H., Randolph J.G., Flanigan L.P. (1990). Cloacal Exstrophy: Individualized Management through a Staged Surgical Approach. J. Pediatr. Surg..

[48] Lee T., Roth E., Shukla A., Gupta N., Lee R., Kryger J., Groth T., Canning D., Mitchell M., Weiss D. (2023). Pelvic Ectopic Kidney Prevalence and Pressure Changes During Cloacal Exstrophy (Omphalocele-Exstrophy-Imperforate Anus-Spinal Defects Syndrome) Closure. Urology.

[49] Mathews R.I., Perlman E., Marsh D.W., Gearhart J.P. (1999). Gonadal Morphology in Cloacal Exstrophy: Implications in Gender Assignment. BJU Int..

[50] Benz K., Maruf M., Hatheway C., Kasprenski M., Jayman J., Docimo S., Schneck F., Gearhart J. (2018). The Intravesical Phallus in Patients with Cloacal Exstrophy: An Embryologic Conundrum. J. Pediatr. Urol..

[51] Tomaszewski J.J., Smaldone M.C., Cannon G.M., Schneck F.X., Hackam D.J., Docimo S.G. (2012). Cloacal Exstrophy Variant with Intravesical Phallus: Further Description of Anatomy and Implications for Gender Reassignment. J. Pediatr. Urol..

[52] Suson K.D., Preece J., Di Carlo H.N., Baradaran N., Gearhart J.P. (2016). Complexities of Müllerian Anatomy in 46XX Cloacal Exstrophy Patients. J. Pediatr. Adolesc. Gynecol..

[53] Judy B.F., Materi J., Lee R.P., Tracz J.A., Patel J., Weber-Levine C., Crigger C., Mistry P., Gearhart J.P., Jackson E.M. (2023). Spinal Dysraphism in Exstrophy: A Single-Center Study of a 39-Year Prospective Database. J. Neurosurg. Pediatr..

[54] Kumar N., Chatur C., Balani A., Bisharat M., Tahir Z., Johal N., Sudhakar S., Cuckow P., Thompson D.N.P., Mankad K. (2021). Patterns of Spinal Cord Malformation in Cloacal Exstrophy. J. Neurosurg. Pediatr..

[55] Suson K.D., Colombani P.M., Jallo G.I., Gearhart J.P. (2013). Intracranial Anomalies and Cloacal Exstrophy--Is There a Role for Screening?. J. Pediatr. Surg..

[56] Haffar A., Hirsch A.M., Crigger C.B., Harris T.G.W., Haney N.M., Galansky L.B., Nasr I.W., Sponseller P.D., Gearhart J.P. (2023). Multi-Staged vs Single-Staged Pelvic Osteotomy in the Modern Treatment of Cloacal Exstrophy: Bridging the Gap. J. Pediatr. Surg..

[57] Mundy A., Kushare I., Jayanthi V.R., Samora W.P., Klingele K.E. (2016). Incidence of Hip Dysplasia Associated With Bladder Exstrophy. J. Pediatr. Orthop..

[58] Bischoff A., Levitt M.A., Breech L., Peña A. (2013). Covered Cloacal Exstrophy--a Poorly Recognized Condition: Hints for a Correct Diagnosis. J. Pediatr. Surg..

[59] Weiss D.A., Borer J.G., Lee R.S., Kryger J.V., Roth E.B., Groth T.W., Shukla A.R., Mitchell M.E., Canning D.A., Mattei P. (2022). Bladder and Cloacal Exstrophy. Fundamentals of Pediatric Surgery.

[60] Holcomb G.W., Murphy J.P., St Peter S.D. (2019). Ashcraft’s Pediatric Surgery.

[61] Jayman J., Tourchi A., Feng Z., Trock B.J., Maruf M., Benz K., Kasprenski M., Baumgartner T., Friedlander D., Sponseller P. (2019). Predictors of a Successful Primary Bladder Closure in Cloacal Exstrophy: A Multivariable Analysis. J. Pediatr. Surg..

[62] Thomas J.C., DeMarco R.T., Pope J.C., Adams M.C., Brock J.W. (2007). First Stage Approximation of the Exstrophic Bladder in Patients with Cloacal Exstrophy--Should This Be the Initial Surgical Approach in All Patients?. J. Urol..

[63] Hurwitz R.S., Manzoni G.A., Ransley P.G., Stephens F.D. (1987). Cloacal Exstrophy: A Report of 34 Cases. J. Urol..

[64] Mathews R., Jeffs R.D., Reiner W.G., Docimo S.G., Gearhart J.P. (1998). Cloacal Exstrophy--Improving the Quality of Life: The Johns Hopkins Experience. J. Urol..

[65] Haney N.M., Crigger C.B., Sholklapper T., Mudalegundi S., Griggs-Demmin A., Nasr I.W., Sponseller P.D., Gearhart J.P. (2023). Pelvic Osteotomy in Cloacal Exstrophy: A Changing Perspective. J. Pediatr. Surg..

[66] Jayman J., Michaud J., Maruf M., Trock B.J., Kasprenski M., Sponseller P., Gearhart J. (2019). The Dual-Staged Pathway for Closure in Cloacal Exstrophy: Successful Evolution in Collaborative Surgical Practice. J. Pediatr. Surg..

[67] Bischoff A., Brisighelli G., Levitt M.A., Peña A. (2014). The “Rescue Operation” for Patients with Cloacal Exstrophy and Its Variants. Pediatr. Surg. Int..

[68] Husmann D.A., McLorie G.A., Churchill B.M., Ein S.H. (1988). Management of the Hindgut in Cloacal Exstrophy: Terminal Ileostomy versus Colostomy. J. Pediatr. Surg..

[69] Maruf M., Kasprenski M., Jayman J., Goldstein S.D., Benz K., Baumgartner T., Gearhart J.P. (2018). Achieving Urinary Continence in Cloacal Exstrophy: The Surgical Cost. J. Pediatr. Surg..

[70] Hisamatsu E., Nakagawa Y., Sugita Y. (2014). Vaginal Reconstruction in Female Cloacal Exstrophy Patients. Urology.

[71] Mathews R. (2011). Achieving Urinary Continence in Cloacal Exstrophy. Semin. Pediatr. Surg..

[72] Tirrell T.F., Demehri F.R., Lillehei C.W., Borer J.G., Warf B.C., Dickie B.H. (2022). Hindgut Duplication in an Infant with Omphalocele-Exstrophy-Imperforate Anus-Spinal Defects (OEIS) Complex. Eur. J. Pediatr. Surg. Rep..

[73] Friedlander D.A., Di Carlo H.N., Sponseller P.D., Gearhart J.P. (2017). Complications of Bladder Closure in Cloacal Exstrophy: Do Osteotomy and Reoperative Closure Factor In?. J. Pediatr. Surg..

[74] Ben-Chaim J., Peppas D.S., Sponseller P.D., Jeffs R.D., Gearhart J.P. (1995). Applications of Osteotomy in the Cloacal Exstrophy Patient. J. Urol..

[75] Shah B.B., Di Carlo H., Goldstein S.D., Pierorazio P.M., Inouye B.M., Massanyi E.Z., Kern A., Koshy J., Sponseller P., Gearhart J.P. (2014). Initial Bladder Closure of the Cloacal Exstrophy Complex: Outcome Related Risk Factors and Keys to Success. J. Pediatr. Surg..

[76] Casey J.T., Chan K.H., Hasegawa Y., Large T., Judge B., Kaefer M., Misseri R., Rink R.C., Ueoka K., Cain M.P. (2017). Long-Term Follow-up of Composite Bladder Augmentation Incorporating Stomach in a Multi-Institutional Cohort of Patients with Cloacal Exstrophy. J. Pediatr. Urol..

[77] Yano K., Sugita K., Kawano T., Murakami M., Harumatsu T., Onishi S., Yamada K., Muto M., Ieiri S., Kubota M. (2023). The Clinical Features of Patients Who Underwent Bladder Augmentation of Cloacal Exstrophy and Their Functional Outcomes: The Results of a Nationwide Survey in Japan. Pediatr. Surg. Int..

[78] Musleh L., Privitera L., Paraboschi I., Polymeropoulos A., Mushtaq I., Giuliani S. (2022). Long-Term Active Problems in Patients with Cloacal Exstrophy: A Systematic Review. J. Pediatr. Surg..

[79] Levitt M.A., Mak G.Z., Falcone R.A., Peña A. (2008). Cloacal Exstrophy--Pull-through or Permanent Stoma? A Review of 53 Patients. J. Pediatr. Surg..

[80] Srinivas S., Knaus M.E., Avansino J.R., Badillo A., Calkins C.M., Dickie B.H., Durham M.M., Fuller M.K., Ralls M.W., Reeder R.W. (2024). Outcomes From Colonic Pull-Through for Cloacal Exstrophy Differ by Colon Length: A Multi-Institutional Study. J. Pediatr. Surg..

[81] Gordetsky J., Joseph D.B. (2015). Cloacal Exstrophy: A History of Gender Reassignment. Urology.

[82] Gooren L. (2006). The Biology of Human Psychosexual Differentiation. Horm. Behav..

[83] Reiner W.G. (2004). Psychosexual Development in Genetic Males Assigned Female: The Cloacal Exstrophy Experience. Child. Adolesc. Psychiatr. Clin. N. Am..

[84] Reiner W.G., Gearhart J.P. (2004). Discordant Sexual Identity in Some Genetic Males with Cloacal Exstrophy Assigned to Female Sex at Birth. N. Engl. J. Med..

[85] Mukherjee B., McCauley E., Hanford R.B., Aalsma M., Anderson A.M. (2007). Psychopathology, Psychosocial, Gender and Cognitive Outcomes in Patients with Cloacal Exstrophy. J. Urol..

[86] Diamond D.A., Burns J.P., Mitchell C., Lamb K., Kartashov A.I., Retik A.B. (2006). Sex Assignment for Newborns with Ambiguous Genitalia and Exposure to Fetal Testosterone: Attitudes and Practices of Pediatric Urologists. J. Pediatr..

[87] Diamond D.A., Burns J.P., Huang L., Rosoklija I., Retik A.B. (2011). Gender Assignment for Newborns with 46XY Cloacal Exstrophy: A 6-Year Followup Survey of Pediatric Urologists. J. Urol..

[88] Harris K.T., Villela N.A., Alam R., Wu W.J., Artigas P., DiCarlo H.N., Gearhart J.P. (2023). The Exstrophy Experience: A National Survey Assessing Urinary Continence, Bladder Management, and Oncologic Outcomes in Adults. J. Pediatr. Urol..

[89] Haney N.M., Gearhart J.P. (2022). Commentary on Long-Term Active Problems in Patients with Cloacal Exstrophy: A Systematic Review. J. Pediatr. Surg..

[90] Fuchs M.E., Ahmed M., Dajusta D.G., Gargollo P., Kennedy U.K., Rosoklija I., Strine A.C., Whittam B., Yerkes E., Szymanski K.M. (2023). Urinary and Bowel Management in Cloacal Exstrophy: A Long-Term Multi-Institutional Cross-Sectional Study. J. Pediatr. Urol..

[91] Mathews R.I., Gan M., Gearhart J.P. (2003). Urogynaecological and Obstetric Issues in Women with the Exstrophy-Epispadias Complex. BJU Int..

[92] Goldstein S.D., Inouye B.M., Reddy S., Lue K., Young E.E., Abdelwahab M., Grewal M., Wildonger S., Stec A.A., Gearhart J.P. (2016). Continence in the Cloacal Exstrophy Patient: What Does It Cost?. J. Pediatr. Surg..

[93] Fullerton B.S., Sparks E.A., Hall A.M., Chan Y.-M., Duggan C., Lund D.P., Modi B.P., Jaksic T., Hendren W.H. (2016). Growth Morbidity in Patients with Cloacal Exstrophy: A 42 Year Experience. J. Pediatr. Surg..

[94] Taskinen S., Rintala R., Mäkitie O. (2008). Bone Health in Patients with Cloacal Exstrophy and Persistent Cloaca after Bladder Augmentation. J. Pediatr. Surg..

[95] Gezer A., Guralp O., Yesilbas C., Madazli R. (2011). Spontaneous Pregnancy and Birth with Corrected Cloacal Exstrophy. Acta Obstet. Gynecol. Scand..

[96] Reppucci M.L., Alaniz V.I., Wehrli L.A., de La Torre L., Wood D., Wilcox D.T., Appiah L.C., Peña A., Bischoff A. (2023). Reproductive and Family Building Considerations for Female Patients with Anorectal And Urogenital Malformations. J. Pediatr. Surg..

[97] Dy G.W., Willihnganz-Lawson K.H., Shnorhavorian M., Delaney S.S., Amies Oelschlager A.-M., Merguerian P.A., Grady R., Miller J.L., Cheng E.Y. (2015). Successful Pregnancy in Patients with Exstrophy-Epispadias Complex: A University of Washington Experience. J. Pediatr. Urol..

[98] Seat M., Boxwalla M., Hough A., Goodwin G. (2022). A Case of Congenital Cloacal Exstrophy/Omphalocele-Exstrophy-Imperforate Anus-Spinal Defects Syndrome and a Successful Pregnancy. Clin. Exp. Reprod. Med..

